# Drotrecogin alfa (activated) in patients with severe sepsis presenting with purpura fulminans, meningitis, or meningococcal disease: a retrospective analysis of patients enrolled in recent clinical studies

**DOI:** 10.1186/cc3538

**Published:** 2005-05-17

**Authors:** Jean-Louis Vincent, Simon Nadel, Demetrios J Kutsogiannis, RT Noel Gibney, S Betty Yan, Virginia L Wyss, Joan E Bailey, Carol L Mitchell, Samiha Sarwat, Stephen M Shinall, Jonathan M Janes

**Affiliations:** 1 Head, Department of Intensive Care, University of Brussels (Erasme Hospital), Brussels, Belgium; 2 Consultant in Paediatric Intensive Care, Department of Paediatrics, Imperial College London (St. Mary's Hospital), London, UK; 3 Assistant Professor, Department of Public Health Sciences, Division of Critical Care Medicine, University of Alberta (Royal Alexandra Hospital), Edmonton, Alberta, Canada; 4 Professor, Division of Critical Care Medicine, University of Alberta (University of Alberta Hospital), Edmonton, Alberta, Canada; 5 Research Fellow, Lilly Research Laboratories, Indianapolis, IN, USA; 6 Associate Consultant, Project Management, Lilly Research Laboratories, Indianapolis, IN, USA; 7 Clinical Development Associate, Lilly Research Laboratories, Indianapolis, IN, USA; 8 Associate Global Medical Information Consultant, Lilly Research Laboratories, Indianapolis, IN, USA; 9 Statistician, Lilly Research Laboratories, Indianapolis, IN, USA; 10 Scientific Communications Associate, Lilly Research Laboratories, Indianapolis, IN, USA; 11 Medical Advisor, Lilly Research Centre, Erl Wood Manor, Windlesham, Surrey, UK

## Abstract

**Introduction:**

We report data from adult and pediatric patients with severe sepsis from studies evaluating drotrecogin alfa (activated) (DrotAA) and presenting with purpura fulminans (PF), meningitis (MEN), or meningococcal disease (MD) (PF/MEN/MD). Such conditions may be associated with an increased bleeding risk but occur in a relatively small proportion of patients presenting with severe sepsis; pooling data across clinical trials provides an opportunity for improving the characterization of outcomes.

**Methods:**

A retrospective analysis of placebo-controlled, open-label, and compassionate-use trials was conducted. Adult patients received infusions of either DrotAA or placebo. All pediatric patients (<18 years old) received DrotAA. 189 adult and 121 pediatric patients presented with PF/MEN/MD.

**Results:**

Fewer adult patients with PF/MEN/MD met cardiovascular (68.3% versus 78.8%) or respiratory (57.8% versus 80.5%) organ dysfunction entry criteria than those without. DrotAA-treated adult patients with PF/MEN/MD (*n *= 163) had an observed 28-day mortality rate of 19.0%, a 28-day serious bleeding event (SBE) rate of 6.1%, and an intracranial hemorrhage (ICH) rate of 4.3%. Six of the seven ICHs occurred in patients with MEN (three of whom were more than 65 years old with a history of hypertension). DrotAA-treated adult patients without PF/MEN/MD (*n *= 3,088) had an observed 28-day mortality rate of 25.5%, a 28-day SBE rate of 5.8%, and an ICH rate of 1.0%. In contrast, a greater number of pediatric patients with PF/MEN/MD met the cardiovascular organ dysfunction entry criterion (93.5% versus 82.5%) than those without. DrotAA-treated PF/MEN/MD pediatric patients (*n *= 119) had a 14-day mortality rate of 10.1%, an SBE rate of 5.9%, and an ICH rate of 2.5%. DrotAA-treated pediatric patients without PF/MEN/MD (*n *= 142) had a 14-day mortality rate of 14.1%, an SBE rate of 9.2%, and an ICH rate of 3.5%.

**Conclusion:**

DrotAA-treated adult patients with severe sepsis presenting with PF/MEN/MD had a similar SBE rate, a lower observed 28-day mortality rate, and a higher observed rate of ICH than DrotAA-treated patients without PF/MEN/MD. DrotAA-treated pediatric patients with severe sepsis with PF/MEN/MD may differ from adults, because all three outcome rates (SBE, mortality, and ICH) were lower in pediatric patients with PF/MEN/MD.

## Introduction

Despite the development of novel anti-infective therapies and improved patient management, severe sepsis remains a serious healthcare concern with an unacceptable mortality rate and an increasing incidence rate that has resulted in a significant economic and societal burden [1-3]. Although there has been a theoretical basis for blocking the excessive inflammatory response evoked during sepsis, so far such approaches have not led to the licensing of new compounds for the treatment of severe sepsis [4]. The contribution of coagulopathy to the pathophysiology of sepsis has become more widely understood [4-7] and has increased the interest in compounds that modulate the coagulation cascade such as antithrombin, tissue factor pathway inhibitor, and activated protein C [8-10]. Although several of these agents have been evaluated in large clinical trials, only recombinant human activated protein C (drotrecogin alfa (activated) (DrotAA; Xigris^®^); Eli Lilly and Company, Indianapolis, IN, USA] has been found to reduce 28-day all-cause mortality. DrotAA has been approved for treatment of adult patients with severe sepsis in more than 50 countries: in the USA, it is indicated for the reduction of mortality in adult patients with severe sepsis (sepsis associated with acute organ dysfunction) who have a high risk of death (for example, an Acute Physiology and Chronic Health Evaluation II (APACHE II) score of 25 or more); in the European Union, it is indicated (when added to best standard care) for the treatment of adult patients with severe sepsis and multiple organ failure.

Like endogenous activated protein C, DrotAA is a regulator of coagulation, fibrinolysis, and inflammation [11]. Consistent with its anticoagulant and profibrinolytic activity is its association with an increased incidence of serious bleeding events (SBEs), particularly in patients predisposed to bleeding [9,12]. Although the bleeding risk is modest, questions have arisen about treatment with DrotAA in patients predisposed to bleeding such as those with disseminated intravascular coagulation. In this relatively prevalent (about 30%) subpopulation of sepsis patients [13], retrospective analysis of data derived from a single trial recently demonstrated a favorable benefit-risk profile for DrotAA [14].

To examine additional safety information in smaller subgroups of patients, it is often helpful to pool experience across studies. Purpura fulminans (PF), with its attendant consumptive coagulopathy, and meningitis (MEN), with its attendant risk of intracranial hemorrhage (ICH), are two conditions seen in septic patients that, although not rare, are much less prevalent than disseminated intravascular coagulation [15-17]. Because both coagulopathy and MEN are sequelae of *Neisseria meningitidis *infection, patients with meningococcal disease (MD) may represent an additional population predisposed to bleeding complications [18,19]. Owing in part to the low incidence of PF, MEN, and MD (3% or less) in sepsis studies, limited data are available characterizing SBEs in septic patients with these conditions [15,19-21]. Uncertainty about the true SBE rates in the sepsis population confounds the interpretation of safety data from the few case reports describing the use of DrotAA in patients with PF, MEN, or MD [22-28].

The recent completion of several clinical studies evaluating DrotAA as an adjunctive treatment in severe sepsis affords an opportunity to improve our understanding of patients presenting with clinical signs and symptoms of PF, MEN, or MD. Here we report the baseline characteristics, mortality outcomes, and observed incidence rates of serious adverse events (especially SBEs and ICHs) in patients with and without PF, MEN, or MD.

## Materials and methods

### Data collection

Data were extracted from four clinical studies investigating DrotAA in adult and pediatric patients with severe sepsis. A database of 4,360 patients (4,096 adult, 264 pediatric) was assembled and, using retrospectively defined criteria, 310 patients (189 adult, 121 pediatric) with signs and symptoms of PF, MEN, or MD were identified, most of whom received DrotAA (165 adult, 121 pediatric). The studies pooled included, first, one multicenter, placebo-controlled, randomized, double-blind, phase 3 trial ('PROWESS', 1,690 adult patients enrolled; 850 DrotAA-treated, 840 placebo); second, one multicenter, open-label phase 3b study ('ENHANCE', 2,378 adult and 188 pediatric patients enrolled); third, one phase 2b open-label pediatric trial (EVAO, 83 patients enrolled); and fourth, one open-label compassionate-use study (EVAS, 28 adult and 14 pediatric patients enrolled) [9,29,30]. Pediatric patients (*n *= 21) enrolled in the dose-escalation phase of EVAO were not included in the present investigation [29]. Study investigators adhered to good clinical practices and ethical principles as stated in the Declaration of Helsinki of 1975, revised in 1983.

### Trial inclusion and exclusion criteria

PROWESS and ENHANCE, as detailed previously, used similar inclusion criteria: proven or suspected infection; three or more signs of systemic inflammatory response syndrome (SIRS) (two or more signs of SIRS for pediatric patients); and evidence of one or more sepsis-induced organ dysfunctions (cardiovascular, respiratory, renal, hematologic, or metabolic acidosis) [9,30]. In comparison with PROWESS, the ENHANCE study design resulted in a longer time between the identification of acute organ dysfunction and initiation of the study drug. The EVAO study enrolled pediatric patients with severe sepsis and used the following inclusion criteria: proven or suspected infection; two or more signs of SIRS within 24 hours of study entry; and evidence of one or more sepsis-induced organ dysfunctions (cardiovascular, respiratory, renal, or hematologic) [29]. The original protocol for EVAO allowed enrollment on the basis of either cardiovascular or respiratory organ dysfunction but was subsequently amended to include renal and hematologic dysfunction in addition. The single inclusion criterion for the EVAS study was a clinical diagnosis of PF. Exclusion criteria were largely similar between trials: body weight more than 135 kg (and less than 3 kg for pediatric patients); platelet count less than 30,000/mm^3 ^(the EVAS study did not exclude patients on the basis of platelet count); congenital or acquired conditions that increase the risk of serious bleeding; moribund state and presumed imminent death (within 24 hours for PROWESS, ENHANCE, and EVAO trials; within 6 hours for EVAS); and recent pharmacologic intervention that might induce a hypocoagulable state [9,29,30].

### Patient selection and definitions

PF, MEN, and MD were not prospectively defined subgroups in the four trials, with the exception that a diagnosis of PF was required for enrollment in the compassionate-use study. A two-step identification process was developed for this retrospective analysis. Both study case report forms and investigator reports of serious adverse events were interrogated for medical and microbiological terms associated with PF, MEN, or MD. Data from patients identified in step one were then reviewed in detail and, on the basis of predefined selection criteria, patients were assigned to one or more of the following groups: PF, MEN, and MD. Because a prospective diagnosis of PF was required for enrollment in EVAS, all these patients were included in the PF group.

In a similar manner to previous retrospective analyses [31-33], the diagnosis of MEN was based on the following criteria: cerebrospinal fluid (CSF) findings consistent with MEN (positive CSF culture, leukocytosis, or positive CSF Gram stain); a clinical picture consistent with MEN (meningismus, headache, stiff neck, photophobia) together with the positive culture of a MEN-associated microorganism; or clinical diagnosis of MEN listed in the case comments. The diagnosis of PF was based on clinical diagnosis or purpuric rash, necrosis of digits, or gangrene recorded in the case comments or serious adverse event reports. A diagnosis of MD was based on clinical diagnosis in case comments or the identification of *N. meningitidis *in CSF or blood (positive culture, positive Gram stain, or other techniques such as polymerase chain reaction).

Bleeding events reported as serious adverse events (namely SBEs) included fatal or life-threatening events (patient at risk of death at the time of event occurrence), ICHs, or events associated with the following transfusion requirements: at least 3 units of packed red blood cells (RBCs) per day for two consecutive days (adult patients and pediatric patients 12 years to less than 18 years old); at least 20 ml of packed RBCs per kilogram per 24 hours (pediatric patients less than 1 year old); at least 10 ml of packed RBCs per kilogram per 24 hours (pediatric patients 1 year to less than 12 years old).

### Drug administration

Adult and pediatric patients were to receive an intended 96-hour continuous infusion of DrotAA (24 μg kg^-1 ^h^-1^); EVAS patients could have received up to an 168-hour continuous infusion of DrotAA (24 μg kg^-1 ^h^-1^). In PROWESS, placebo patients received either 0.1% albumin or saline. No pediatric patients received placebo.

### Statistical analysis

Data were extracted from validated clinical trial databases. All calculations were derived with SAS version 8.2 (SAS Institute, Inc., Cary, NC, USA). Continuous data were summarized by means of measures of central tendency and dispersion. Categorical data were summarized with incidence rates and counts. All analyses were exploratory and descriptive; no adjusted statistical analyses of event rates were performed across clinical trials, patient groups, or treatment groups. Twenty-eight-day mortality rates were calculated for adult patients. Pediatric mortality rate calculations were limited to 14-day endpoints because of differences in study design. Mortality and SBE rates are presented with 95% confidence intervals (CIs) generated with the exact CI method. Unadjusted odds ratios with 95% CIs were generated for the effect of diagnostic group membership (with or without PF, MEN, or MD) on mortality.

## Results

### Adult patients

**Figure 1:**
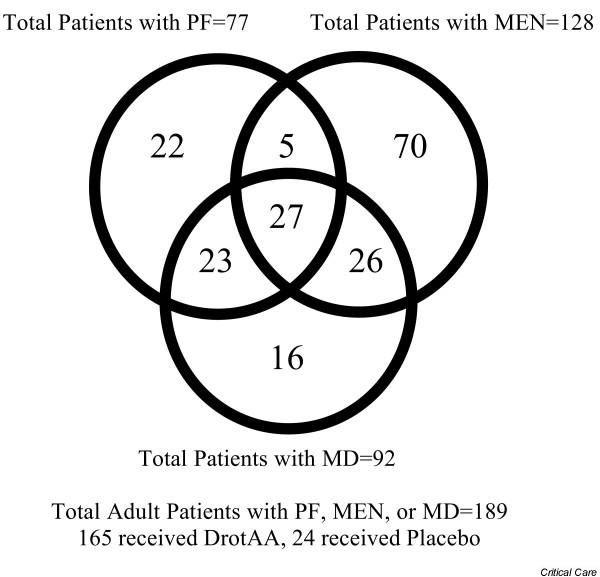
Venn diagram of adult patient distribution by disease category.

One hundred eighty-nine (4.6%) of the total 4,096 adult patients with severe sepsis were identified as having PF, MEN, or MD. Because patients could be classified as having multiple diagnoses, there was substantial overlap between patient groups (Fig. 1). Most of the 189 patients were derived from either the ENHANCE (DrotAA, *n *= 111) or PROWESS (DrotAA, *n *= 26; PLC, *n *= 24) trials, and the remaining patients were enrolled in the EVAS compassionate-use study (DrotAA, *n *= 28).

Baseline characteristics of adult patients with severe sepsis presenting with PF, MEN, or MD are shown in Table 1. Patients with PF, MEN, or MD were younger, with less sepsis-associated organ dysfunction and fewer underlying comorbidities but with more thrombocytopenia. Less time elapsed from first organ dysfunction to the start of DrotAA treatment in patients with PF, MEN, or MD (mean 18.3 hours) than in those without (mean 22.6 hours). PF patients had the shortest mean time to treatment (mean 13.5 hours) and the lowest median baseline protein C level (30% of normal adult pooled plasma level). Although ENHANCE potentially allowed a longer window than PROWESS from first organ dysfunction to the start of treatment, the median time-to-DroAA treatment for patients with PF, MEN, or MD from ENHANCE was 15.7 hours; for those treated with DroAA from PROWESS it was 18.6 hours.

Predominant etiologic pathogens for patients with PF (67 of 77 (87%) had a culture result available) were *N. meningitidis *(50 of 67; 75%) and *Streptococcus pneumoniae *(11 of 67; 16%). Similarly, for patients with MEN the most common pathogens (111 of 128 (87%) had a culture result available) were *N. meningitidi*s (51 of 111; 46%) and *S. pneumoniae *(37 of 111; 33%). For the 24 placebo-treated patients with PF, MEN, or MD, the baseline mean APACHE II score was 26.0 (SD 8.3) and the baseline median number of organ dysfunctions was two.

Table 2 summarizes 28-day all-cause mortality and safety data for adults with PF, MEN, or MD treated with DrotAA. The observed mortality rates for patients with and without PF, MEN, or MD were 19.0% and 25.5%, respectively. The unadjusted odds ratio for patients with versus those without PF, MEN, or MD was 0.69 (95% CI 0.44 to 1.03). Although not shown in Table 2, the mortality rate for placebo-treated patients with PF, MEN, or MD (all from the PROWESS clinical trial) was 25.0% (6 of 24).

During the DrotAA infusion period (defined as the duration of DrotAA infusion plus one full calendar day), rates of total SBEs were similar between patients with and without PF, MEN, or MD (3.7% versus 3.2%), including both fatal (0.6% versus 0.4%) and life-threatening (1.2% versus 1.4%) events. SBE rates during the 28-day study period were also similar between patients with and without PF, MEN, or MD (6.1% versus 5.8%).

ICH rates seemed to differ between the two main diagnostic groupings. Among the DrotAA-treated patients with PF, MEN, or MD, two-thirds (4 of 6) of the SBEs observed during the infusion period were ICHs (ICH rate 2.5%; 4 of 163), whereas 13 of 100 SBEs were ICHs (ICH rate 0.4%; 13 of 3,088) in patients without PF, MEN, or MD. The ICH rate for the 28-day study period was 4.3% for patients with PF, MEN, or MD and 1.0% for patients without PF, MEN, or MD. Among the 24 placebo-treated patients with PF, MEN, or MD from PROWESS, only one SBE (an ICH in a patient with PF and pneumococcal sepsis) was reported.

Because DrotAA has been approved for the treatment of adults with severe sepsis with two or more organ dysfunctions (for example in the European Union) or at high risk of death in the USA (for example an APACHE II score of 25 or more), mortality as well as SBEs for DrotAA-treated patients are also presented by baseline disease severity in Table 3. Baseline APACHE II and organ dysfunction data were available for only 137 of the total 163 DrotAA-treated adults with PF, MEN, or MD; it was not collected for the 26 DrotAA-treated adults with PF, MEN, OR MD from the compassionate-use open-label trial EVAS. DrotAA-treated adults with PF, MEN, or MD with either a baseline APACHE II score of 25 or more or with at least two baseline organ dysfunctions still had lower 28-day mortality rates than those in the high-severity subgroups without PF, MEN, or MD. Observed serious bleeding rates (infusion as well as 28-day) in the stratified groups were similar to all-event rates.

Table 4 (each column represents data for one patient) summarizes disease categories, baseline disease severity scores, and organ failure assessment scores for the 10 PF, MEN, or MD patients experiencing an SBE during the 28-day study period. All four ICHs during infusion and six of seven ICHs during the 28-day study period occurred in patients with MEN. Nearly half (three of seven) were observed in patients over 65 years old with a history of hypertension. Two of the four ICHs observed during the infusion period were associated with platelet counts less than 80,000/mm^3 ^on the day before the event.

### Pediatric patients

**Figure 2:**
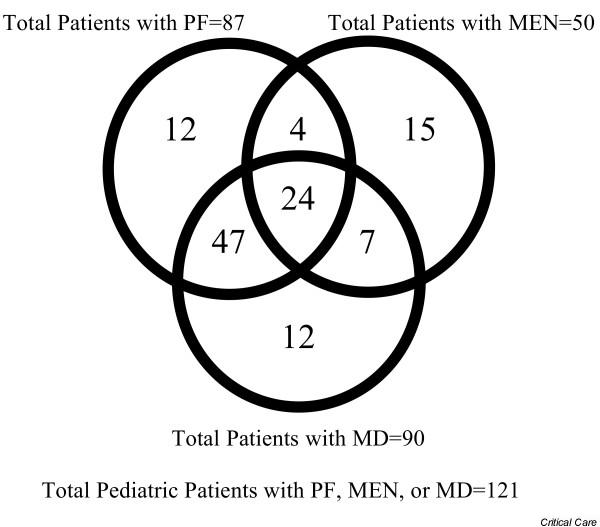
Venn diagram of pediatric patient distribution by disease category.

Of the 264 pediatric patients with severe sepsis, 121 (45.8%) were identified as having PF, MEN, or MD. As shown in Fig. 2, substantial overlap between these patient groups was observed. About 67% (81 of 121) of the patients originated from the pediatric arm of the ENHANCE trial, whereas the remaining 33% were enrolled in either the EVAO (*n *= 26) or EVAS (*n *= 14) studies.

Table 5 shows the baseline characteristics of pediatric patients with PF, MEN, or MD. Patients with PF, MEN, or MD were more likely to require vasopressor support but were less likely to receive ventilator support than those without PF, MEN, or MD. As in adults, DrotAA treatment began sooner after the first organ dysfunction for patients with PF, MEN, or MD (mean 13.0 hours) than in those without (mean 22.3 hours). Among pediatric patients with MEN (42 of 50 (84%) had a culture result available), the most common etiologic pathogens were *N. meningitidis *(31 of 42; 74%) and Group B streptococci (7 of 42; 17%). *N. meningitidis *(68 of 71; 96%) predominated in PF patients (71 of 87 (82%) had a culture result available).

Table 6 summarizes 14-day all-cause mortality and safety data for pediatric patients with severe sepsis with PF, MEN, or MD treated with DrotAA. Patients with PF, MEN, or MD had a lower observed mortality rate than patients without (10.1% versus 14.1%). The unadjusted odds ratio for patients with versus those without PF, MEN, or MD was 0.68 (95% CI 0.29 to 1.60).

During DrotAA infusion, the observed SBE rate was lower for patients with PF, MEN, or MD than for those without PF, MEN, or MD (1.7% versus 7.0%). Furthermore, there were no instances of ICH in patients with PF, MEN, or MD during DrotAA infusion, whereas patients without PF, MEN, or MD had an observed ICH rate of 1.4%. SBE rates for the entire study period were more equivalent between patient groups (with PF, MEN, or MD, 5.9%; without, 9.2%). The reported ICH rate during the study period was also similar between patients with and without PF, MEN, or MD (2.5% versus 3.5%).

Table 7 provides detailed information for pediatric patients experiencing an SBE during the study period. All seven SBEs occurred in patients with PF or MD, and five patients had signs and symptoms consistent with both diagnoses. All patients experiencing an SBE had baseline platelet counts of less than 75,000/mm^3 ^(four patients had platelet counts of 30,000/mm^3 ^or less). Four of the six patients for whom data were available also had a baseline activated partial thromboplastin time of more than 100 s.

## Discussion

Because most adult and all pediatric patients with PF, MEN, or MD in this database were from open-label studies, the ability to make comparisons with a placebo group is limited. In view of the clinical overlap between PF, MEN, and MD, we considered these diagnoses collectively as well as individually. This approach is further supported by the likelihood that, given the retrospective nature of this study, it might not have been possible to complete a full clinical classification of all patients.

Incidence rates of PF, MEN, and MD in patients with severe sepsis are not widely available for comparison. In this analysis fewer than 5% (189 of 4,096) of adult patients with severe sepsis were identified as having PF, MEN, or MD, a finding similar to epidemiological analyses reporting a MEN incidence rate of 3.0% [21]. PF, MEN, and MD were much more prevalent among pediatric patients with severe sepsis with 46% (121 of 264) being diagnosed with or having signs or symptoms of PF, MEN, or MD. PF has been reported in 10 to 20% of patients with MD [34]. In our sample about 54% (50 of 92) of adult MD patients and 79% (71 of 90) of pediatric MD patients also had signs and symptoms of PF, although incidence rates might have been inflated by patient and site selection methods in these clinical trials.

There were important differences in demographic and clinical characteristics within and between diagnostic groupings. Collectively, adults with PF, MEN, or MD were younger with fewer underlying comorbidities than those without PF, MEN, or MD. Considered separately, adults with MEN were slightly older and more frequently had pre-existing hypertension or diabetes than adults with PF or MD. However, adult patients with PF and MD had evidence of greater baseline coagulopathy. For example, protein C deficiency was most severe in the adult PF group, followed by the MD group. Pediatric protein C levels were more consistent between the three diagnostic groupings. However, because protein C levels in children are highly dependent on age [35], baseline imbalances in age between the comparator groups potentially confound the interpretation of protein C deficiency. Protein C levels in the adult patients were more in line with the general perception that patients with PF and MD have worse coagulopathy than MEN patients.

The time from the first organ dysfunction to the start of DrotAA treatment differed between those with and without PF, MEN, or MD. A time-to-treatment difference was even more striking when individual diagnoses were examined, because adult patients with PF and MD began DrotAA treatment sooner than all other adult subgroups. A reduced time to DrotAA treatment probably reflects the more marked and unambiguous clinical presentation of PF and MD.

Mortality rates for MEN in the literature vary widely by pathogen and patient age [20,36,37]. For adults and adolescents, reported mortality rates for bacterial MEN range from 11 to 37% [16,17,31-33,38-42]. For children, mortality rates for MEN tend to be closer to 10% but have been reported to be 21% for those also presenting with shock [37,41,43]. The case fatality rate for MD has been reported to be between 8% and 14%, although can be as high as 20% in those less than 1 year of age [3,36,44]. PF has a much wider reported mortality range of 37 to 60% [45-47]. In the present analysis, mortality rates for patients with PF, MEN, or MD were 19.0% for adults and 10.1% for pediatric patients. However, it is difficult to directly compare clinical trial data, potentially confounded by entry and exclusion criteria, with data from epidemiological reports that may comprise a broader spectrum of patients.

There was insufficient evidence (for example small numbers of patients) to suggest that any mortality rate differences were statistically significant; however, the mortality rate for patients with PF, MEN, or MD certainly does not seem higher for patients without such diagnoses or complications. This trend holds also for patients assessed to have a higher risk of death at baseline, by either APACHE II scores of 25 or more or with at least two organ dysfunctions. Importantly, DrotAA treatment did not seem to increase mortality in adult patients with PF, MEN, or MD, because mortality rates for DrotAA-treated and placebo-treated patients with PF, MEN, or MD were 19.0% and 25.0%, respectively. These findings are consistent with previously published reports showing a mortality reduction associated with DrotAA treatment [9,30]. However, patients with PF, MEN, or MD were younger, presented with fewer underlying comorbidities, and began receiving DrotAA sooner after first organ dysfunction than those without PF, MEN, or MD. Because any of these baseline parameters could influence patient outcome, the data presented here must be interpreted with caution.

DrotAA is a recombinant form of an endogenous regulator of coagulation and, consistent with its antithrombotic properties, is associated with an increased risk of SBEs. In trials evaluating DrotAA in adults, SBE rates range from 3.5 to 5.5% for DrotAA-treated patients, compared with 2.0% for placebo controls [9,30]. As a reference, reported SBE rates ranged from 1 to 6% in the placebo arm of other recently completed clinical trials in severe sepsis [8,10,48]; however, SBE definitions may vary between trials, limiting inter-trial comparisons. In this study, DrotAA-treated adults with and without PF, MEN, or MD generally had similar SBE rates (including fatal or life-threatening bleeding) both during the infusion and 28-day study periods. Patients with PF had a higher SBE rate when considered separately, a finding consistent with the greater baseline coagulopathy observed in this group.

Because of its associated morbidity and mortality, ICH is among the most serious of SBEs. In a 6-year retrospective study of intensive care unit patients developing ICH (*n *= 2,198), Oppenheim and colleagues [49] found a spontaneous ICH rate of 0.4% in the critically ill; patients with sepsis accounted for five of nine patients (56%) developing ICH in their report. Other conditions or comorbidities associated with ICH included thrombocytopenia and impaired renal and hepatic function [49]. Central nervous system bleeding events (including ICHs), seen in the placebo arms of large clinical trials in severe sepsis, tend to be about 0.3% or 0.4% [8,10]. By comparison, the ICH rate for DrotAA-treated patients was 0.2% versus 0.1% for placebo controls in the PROWESS clinical trial [9]. Sharshar and colleagues [50] suggest that the rate of ICH might be much higher in patients with septic shock, as post-mortem examination revealed evidence of cerebral hemorrhage in 6 of 23 septic shock patients (26%). However, direct comparison of cerebral hemorrhage incidence between survivors and non-survivors of septic shock was not conducted, and ICH rates may differ between those who do and do not survive septic shock.

Adults with PF, MEN, or MD had a higher ICH rate than those without PF, MEN, or MD, both during DrotAA infusion (2.5% versus 0.4%) and during the 28-day study period (4.3% versus 1.0%). Considered separately, patients with MEN had the highest ICH rates (3.8% during infusion and 5.7% during the study period). Factors other than MEN that may increase the risk of ICH were also present in patients developing ICH. Nearly half (three of seven) of the patients with ICH were more than 65 years old and had pre-existing hypertension. Moreover, thrombocytopenia was evident in two of four patients with ICH during infusion, and two patients had either hepatic or both hepatic and renal organ dysfunction at the time of the ICH event. Using a database composed of a similar sample of patients from the current study, Bernard and colleagues [12] found that almost half of ICH events during DrotAA infusion occurred in patients with MEN or thrombocytopenia. However, in the analysis by Bernard and colleagues, patients with PF, MEN, or MD were not studied as a collective subgroup, nor were comparisons of mortality outcome and safety made with those in patients without PF, MEN, or MD.

The ICH rates reported here are consistent with previous reports of acute bacterial MEN in non-DrotAA-treated patients. In previous reports, ICH incidence ranged from 1 to 9% [16,17,51]. Despite the apparent increased incidence of ICH in adult PF, MEN, or MD patients, the rates of fatal or life-threatening SBEs did not differ markedly between those with and without PF, MEN, or MD. The observed ICH rates for adults with PF, MEN, or MD receiving either placebo or DrotAA during the study period was similar (4.2% versus 4.3%), although the small placebo sample limits conclusions derived from such a comparison. The data suggest that adults with MEN are at increased risk of ICH. However, the quantity of any additional potential risk resulting from DrotAA treatment is not clear from this analysis.

In contrast to the findings in adults, pediatric patients with PF, MEN, or MD had lower SBE and ICH rates than those without, both during the DrotAA infusion and overall study period. Whereas most SBEs occurred during the infusion period (6 of 10) for adult patients with PF, MEN, or MD, for pediatric patients most SBEs occurred during the post-infusion period. A possible explanation of why pediatric Drot-AA treated patients with PF, MEN, or MD had lower ICH rates than their adult counterparts is that they did not have two of the four risk factors (age more than 65 years, pre-existing hypertension, thrombocytopenia, MEN) that seemed to be associated with increased ICH rates in adult DrotAA-treated patients with PF, MEN, or MD.

In addition, the lack of observed ICHs during DrotAA infusion and an ICH rate of 2.5% during the study period for pediatric patients with PF, MEN, or MD are particularly interesting in view of a recent study of recombinant tissue plasminogen activator treatment in children with meningococcal PF (reported ICH rate 8%; 5 of 62) [52]. However, it is difficult to compare open-label clinical trials and observational case studies directly, because patients enrolled in clinical trials might not represent the same spectrum of disease severity observed in observational studies. For example, the mortality rate for pediatric PF, MEN, or MD patients described in our study was 10.1%, compared with 47% for PF patients in the tissue plasminogen activator study [52].

Differences in both mortality and SBE outcomes between adult and pediatric patients with severe sepsis are intriguing and probably reflect differences in microbial etiology, physiology (for example physiologic reserve), associated underlying disease, and treatment strategies. For example, mortality rates for adult and pediatric patients with MEN in this database were 17.9% and 8.3%, respectively. *S. pneumoniae*, associated with higher mortality in MEN patients, was reported in 33% of adult versus 2% of pediatric patients with MEN.

This study has several limitations. Most adult and all pediatric patients with PF, MEN, or MD in this database were enrolled in open-label studies. Correspondingly, few patients received placebo, making comparisons between DrotAA and placebo groups difficult. Another limitation was that patient subgroups (with or without PF, MEN, and MD) were not defined prospectively. Because these patients were identified through retrospective case review and there was not a prospective requirement to collect all of the clinical data needed to make these diagnoses (except for PF in EVAS), it is possible that some patients having PF, MEN, or MD might have been missed or classified as having only one rather than multiple diagnoses. In addition, patient data were combined from clinical trials with similar but non-identical entry criteria. As a result of differences in study design, not all information of interest was collected for each patient. The combination of small sample size and incomplete data sets precludes robust statistical assessment of the impact of DrotAA treatment on either mortality or SBE and ICH incidence in patients with PF, MEN, or MD. As a result, this study does not definitively address reported differences between patient groups defined by either disease category or treatment.

A general limitation extends from comparing data from clinical trials to results obtained from epidemiological studies or case reviews. All patients described here met specific enrollment criteria. Although comparisons with epidemiological data might be of some utility, any inferences should be interpreted with this caveat in mind.

Despite the limitations, this study provides novel information. So far, data on DrotAA use in the management of PF, MEN, or MD have been limited to case reports [22-28]. Four reports noted a positive outcome in 9 of 10 patients and no serious bleeding complications with meningococcal PF [22,24,27,28]. One report documented a subarachnoid hemorrhage and a fatal outcome for a 67-year-old patient with pneumococcal MEN and septicemia [23]. Two other case reports had positive outcomes: one in an adult with pneumococcal sepsis and PF, the other in an adolescent with congenital protein C deficiency and PF. Findings from our study are in line with current case report data and complement it. One advantage of this study is the large cohort of patients hospitalized with a uniform diagnosis of severe sepsis; thus, the number of patients considered here exceeds that regularly examined in observational studies or case series reviews. Another advantage is the use of prospectively defined primary endpoints and serious adverse events to investigate clinical experience and outcome.

Given our study's limitations, no recommendation can be made about the use of DrotAA in patients with severe sepsis presenting with PF, MEN, or MD, despite this group's observed lower mortality than in those patients without PF, MEN, or MD. Information from this study might be of use to clinicians considering DrotAA treatment in PF, MEN, or MD patients with severe sepsis: safety information from the largest cohort of such patients is made available. No obvious connection between severity of illness as indicated by either an APACHE II score of at least 25 or two or more organ dysfunctions and the occurrence of SBEs is suggested.

## Conclusion

Patients with severe sepsis with signs and symptoms of PF, MEN, or MD are generally perceived to be at higher risk of bleeding complications. In this retrospective study, neither adult nor pediatric patients receiving DrotAA and exhibiting signs and symptoms of PF, MEN, or MD had increased serious bleeding rates (including life-threatening or fatal events) compared with patients without PF, MEN, or MD. Adult, but not pediatric, patients with MEN seemed at increased risk for developing ICH. These findings should be borne in mind when considering DrotAA in the management of patients with severe sepsis with evidence of PF, MEN, or MD. DrotAA is approved only for adult patients with severe sepsis at high risk of death (United States Package Insert) or with multiple organ failure (European Union Summary of Product Characteristics). A large ongoing placebo-controlled study evaluating DrotAA treatment in pediatric patients with severe sepsis will permit a more robust analysis of the benefit-risk profile of DrotAA in pediatric patients.

## Key messages

• 	Patients with severe sepsis with signs and symptoms of purpura fulminans (PF), meningitis (MEN), or meningococcal disease (MD) are generally perceived to be at higher risk of bleeding complications.

• 	In this retrospective study, neither adult nor pediatric patients receiving DrotAA and exhibiting signs and symptoms of PF, MEN, or MD had increased serious bleeding rates (including life-threatening or fatal events) compared with patients without PF, MEN, or MD

• 	Adult, but not pediatric, patients with MEN seemed to be at an increased risk for developing intracranial hemorrhage

• 	These findings should be borne in mind when considering DrotAA in the management of adult patients with severe sepsis with evidence of PF, MEN, or MD

• 	DrotAA is not currently approved for treatment of pediatric patients with severe sepsis, and the results of an ongoing placebo controlled study are awaited

## Abbreviations

APACHE = Acute Physiology and Chronic Health Evaluation; CI = confidence interval; CSF = cerebrospinal fluid; DrotAA = drotrecogin alfa (activated); ICH = intracranial hemorrhage; MD = meningococcal disease; MEN = meningitis; PF = purpura fulminans; RBC = red blood cell; SBE = serious bleeding event; SIRS = systemic inflammatory response syndrome.

## Competing interests

SBY, VLW, JEB, CLM, SS, SMS, and JMJ are employees of Eli Lilly and Company. JLV and SN are consultants for Eli Lilly and Company. DJK and RTNG have declared that they have no competing interests.

## Authors' contributions

All authors were involved in discussions regarding the design and objectives of the study. SS provided statistical expertise and constructed the database. JEB, VLW, SBY, SS, and JMJ reviewed case report forms for adverse event, microbiological, and diagnostic details. SBY, CLM, and SMS performed the literature review. Each author either drafted sections of the manuscript or provided critical revision of important intellectual content. All authors read and approved the final manuscript.

## Figures and Tables

**Table 1:** Baseline characteristics of adult severe sepsis patients with purpura fulminans, meningitis, or meningococcal disease

Baseline characteristics^1^	No PF, MEN, or MD *n *= 3,907 (816 PLC)	PF, MEN, or MD *n *= 189 (24 PLC)	PF *n *= 77 (5 PLC)	MEN *n *= 128 (21 PLC)	MD *n *= 92 (10 PLC)
Demographics and disease severity					
Age (years), mean ± SD	60.3 ± 16.5	44.4 ± 19.6	34.6 ± 14.4	48.2 ± 20.4	34.9 ± 16.1
Male (%)	57.8	53.4	58.4	53.1	50.0
Caucasian (%)	86.8	91.0	92.2	91.4	89.1
Organ dysfunctions, median; q1-q3	3.0; 2.0–3.0	2.0; 1.0–4.0	3.0; 2.0–4.0	2.0; 1.0–3.0	2.0; 2.0–4.0
First organ dysfunction to infusion (h), mean ± SD	22.6 ± 13.0	18.3 ± 13.2	13.5 ± 11.8	18.5 ± 13.4	14.9 ± 11.3
APACHE II score, mean ± SD	23.2 ± 7.6	22.6 ± 8.1	22.3 ± 7.8	22.0 ± 7.9	21.9 ± 7.8
GCS score, mean ± SD	12.2 ± 3.8	10.3 ± 4.1	11.8 ± 3.8	9.8 ± 4.0	11.2 ± 4.1
Underlying comorbidities					
Congestive cardiomyopathy (%)	5.7	0	0	0	0
COPD (%)	19.4	3.1	0	4.0	1.3
Diabetes (%)	21.0	9.9	2.0	11.3	5.1
Hypertension (%)	36.7	18.0	6.1	20.2	7.7
Liver disease (%)	2.9	2.5	4.1	2.4	2.6
Cancer (%)	16.3	7.5	2.0	8.1	5.1
Myocardial infarction (%)	11.6	1.9	0	2.4	0
Pancreatitis (%)	3.7	1.2	0	1.6	1.3
Recent trauma (%)	3.9	1.9	4.1	1.6	1.3
Recent surgery (%)	35.5	1.9	4.1	1.6	1.3
Coagulation biomarkers					
Protein C level (%), median; q1-q3	46; 30–64	47; 30–67	30; 20–45	54; 38–74	40; 27–53
Platelet count, median; q1-q3	172; 105–249	91; 45–142	59; 35–95	119; 65–159	70; 34–121
APTT (s), median; q1-q3	41; 34–50	44; 35–55	48; 41–63	40; 34–49	48; 38–62
PT (s), median; q1-q3	18; 15–21	17; 15–22	17; 14–23	17; 15–21	19; 16–23
Cardiovascular and respiratory measures					
Vasopressor (%)	69.6	60.1	76.6	47.2	72.5
Ventilator (%)	79.3	79.8	80.5	76.4	76.9
Cardiovascular study entry criteria (%)	78.8	68.3	77.6	64.5	79.5
Respiratory study entry criteria (%)	80.5	57.8	59.2	54.0	47.4

^1^Patients with missing data were excluded from this analysis. APACHE II, Acute Physiology and Chronic Health Evaluation II; APTT, activated partial thromboplastin time; COPD, chronic obstructive pulmonary disease; GCS, Glasgow Coma Scale; MD, meningococcal disease; MEN, meningitis; PF, purpura fulminans; PLC, placebo; PT, prothrombin time.

**Table 2:** Serious bleeding and mortality rates in adult severe sepsis patients treated with drotrecogin alfa (activated)

Period and type of event^1^	No PF, MEN, or MD (*n *= 3,088)	PF, MEN, or MD (*n *= 163)	PF (*n *= 70)	MEN (*n *= 106)	MD (*n *= 80)
SBEs during infusion					
All events, % (*n*); 95% CI	3.2 (100); 2.6–3.9	3.7 (6); 1.4–7.8	4.3 (3); 1.0–12.0	3.8 (4); 1.0–9.4	3.8 (3); 0.8–10.6
Fatal, % (*n*)	0.4 (12)	0.6 (1)	0	0.9 (1)	0
Life-threatening, % (*n*)	1.4 (43)	1.2 (2)	1.4 (1)	0.9 (1)	0
ICH, % (*n*)	0.4 (13)	2.5 (4)	1.4 (1)	3.8 (4)	2.5 (2)
SBEs over 28 days					
All events, % (*n*); 95% CI	5.8 (178); 5.0–6.6	6.1 (10); 3.0–11.0	8.6 (6); 3.2–17.7	5.7 (6); 2.1–11.9	3.8 (3); 0.8–10.6
Fatal, % (*n*)	0.8 (24)	0.6 (1)	0	0.9 (1)	0
Life-threatening, % (*n*)	2.6 (81)	2.5 (4)	4.3 (3)	1.9 (2)	0
ICH, % (*n*)	1.0 (32)	4.3 (7)	4.3 (3)	5.7 (6)	2.5 (2)
28-day mortality					
Mortality, % (*n*); 95% CI	25.5 (788); 24.0–27.1	19.0 (31); 13.3–26.0	21.4 (15); 12.5–32.9	17.9 (19); 11.2–26.6	8.8 (7); 3.6–17.2

^1 ^Patients lost to follow-up (No PF, MEN, or MD = 3; PF, MEN, or MD = 2) were excluded from this analysis. DrotAA, drotrecogin alfa (activated); ICH, intracranial hemorrhage; MD, meningococcal disease; MEN, meningitis; PF, purpura fulminans; SBE, serious bleeding event.

**Table 3:** Serious bleeding and mortality rates in DrotAA-treated adults by baseline disease severity

Period and type of event	No PF, MEN, or MD *N *= 3,088	PF, MEN, or MD *N *= 137^1^
28-day mortality		
APACHE II		
≥ 25	35.3 (433/1,227)	22.5 (11/49)
<25	19.1 (355/1,861)	12.5 (11/88)
Number of organ failures		
≥ 2	27.3 (693/2,538)	17.8 (18/101)
<2	17.3 (95/550)	11.1 (4/36)
SBEs during infusion		
APACHE II		
≥ 25	3.7 (45/1,227)	4.1 (2/49)
<25	3.0 (55/1,861)	3.4 (3/88)
Number of organ failures		
≥ 2	3.4 (85/2,538)	4.0 (4/101)
<2	2.7 (15/550)	2.8 (1/36)
SBEs, 28-day		
APACHE II		
≥ 25	6.2 (76/1,227)	6.1 (3/49)
<25	5.5 (102/1,861)	4.6 (4/88)
Number of organ failures		
≥ 2	6.0 (151/2,538)	5.0 (5/101)
<2	4.9 (27/550)	5.6 (2/36)

^1^Baseline APACHE II and baseline organ dysfunction data available for 137 patients (not collected for the 26 DrotAA-treated adults from the compassionate-use open-label EVAS trial. APACHE, Acute Physiology and Chronic Health Evaluation; DrotAA, drotrecogin alfa (activated); MD, meningococcal disease; MEN, meningitis; PF, purpura fulminans; SBE, serious bleeding event.

**Table 4:** Characteristics of DrotAA-treated adults with PF, MEN, or MD and experienced a serious bleeding event

Characteristic	During infusion						After infusion			
		
Relative day of event onset^1^	1	1	2	3	5	6	6	7	11	18
Bleeding event										
ICH	No	No	Yes	Yes	Yes	Yes	Yes	Yes	Yes	No
Fatal	No	No	No	No	Yes	No	No	No	Yes	No
DrotAA-related^2^	Yes	No	No	No	Yes	Yes	No	Yes	No	No
Disease category										
PF	Yes	Yes	No	No	No	Yes	Yes	No	Yes	Yes
MEN	No	No	Yes	Yes	Yes	Yes	No	Yes	Yes	No
MD	Yes	No	Yes	No	No	Yes	No	No	No	Yes
Baseline characteristics										
Age (years)	19	20	47	67	73	24	41	77	51	40
Protein C level (%)	11	NA	11	68	55	79	-	52	-	24
Platelet count	38	58	154	102	93	30	136	56	11	51
APTT (s)	198	86.7	65.0	31.1	-	63.0	-	32.0	-	47
PT (s)	58.9	16.6	-	14.1	-	-	16.3	-	2.6	-
Organ failure	5	NA	2	1	3	3	-	1	-	4
APACHE II score	30	NA	20	23	24	26	-	20	-	25
Hypertension	No	No	No	Yes	Yes	No	No	Yes	No	No
SOFA^3^										
Platelet count	38	NA	70	120	181	34	NA	NA	NA	NA
Hematology SOFA score	3	NA	2	1	0	3	NA	NA	NA	NA
Hepatic SOFA score	1	NA	1	0	-	2	NA	NA	NA	NA
Renal SOFA score	2	NA	2	0	0	0	NA	NA	NA	NA

^1^Day 0 is defined as the calendar day on which DrotAA treatment began; ^2^this denotes whether or not the investigator considered the bleeding event to be related to DrotAA treatment; ^3^values reported are those obtained 1 day before the relative onset day of the ICH. Data available only during first 6 days of enrollment in the PROWESS and ENHANCE trials. APACHE II, acute physiology and chronic health evaluation II; APTT, activated partial thromboplastin time; BL, baseline; DrotAA, drotrecogin alfa (activated); ICH, intracranial hemorrhage; MD, meningococcal disease; MEN, meningitis; NA, not available; PC, protein C activity; PF, purpura fulminans; PT, prothrombin time; SOFA, Sequential Organ Failure Assessment. A dash indicates missing data.

**Table 5:** Baseline characteristics of pediatric severe sepsis patients with purpura fulminans, meningitis, or meningococcal disease

Baseline characteristics^1^	No PF, MEN, or MD *N *= 143	PF, MEN, or MD *N *= 121	PF *N *= 87	MEN *N *= 50	MD *N *= 90
Demographics and disease severity					
Age (years), mean ± SD	6.8 ± 6.3	5.6 ± 5.8	6.1 ± 5.9	5.7 ± 6.2	5.9 ± 5.8
Male (%)	46.2	53.7	50.6	52.0	55.9
Caucasian (%)	63.6	86.8	90.8	80.0	92.2
Organ dysfunctions, median; q1-q3	2.0; 1.0–3.0	2.0; 1.0–3.0	2.0; 1.0–3.0	2.0; 1.0–3.0	2.0; 1.0–3.0
First organ dysfunction to infusion (h), mean ± SD	22.3 ± 13.3	13.0 ± 10.0	13.2 ± 9.5	12.3 ± 9.1	13.0 ± 10.2
Coagulation biomarkers					
Protein C level (%), median; q1-q3	38; 24–59	27; 19–38	29; 19–38	24; 16–41	26; 18–35
Platelet count, median; q1-q3	115; 58–205	88; 51–142	85; 42–122	107; 67–178	87; 53–132
APTT (s), median; q1-q3	46; 35–59	57; 41–79	57; 40–82	49; 40–70	57; 40–80
PT (s), median; q1-q3	16; 14–21	20; 15–26	20; 16–26	17; 15–23	20; 16–25
Cardiovascular and respiratory measures					
Vasopressor (%)	70.8	87.4	90.0	81.6	87.5
Ventilator (%)	90.6	80.0	82.9	76.3	79.2
Cardiovascular study entry criteria (%)	82.5	93.5	97.3	90.9	96.2
Respiratory study entry criteria (%)	64.3	34.6	37.0	34.1	30.8

^1^Patients with missing data were excluded from this analysis. APTT, activated partial thromboplastin time; MD, meningococcal disease; MEN, meningitis; PF, purpura fulminans; PT, prothrombin time.

**Table 6:** Serious bleeding and mortality rates in pediatric severe sepsis patients treated with drotrecogin alfa (activated)

Period and type of event^1^	No PF, MEN, or MD *N *= 142	PF, MEN, or MD *N *= 119	PF *N *= 85	MEN *N *= 48	MD *N *= 88
Serious bleeding events during infusion					
All events, % (*n*); 95% CI	7.0 (10); 3.4–12.6	1.7 (2); 0.2–6.0	2.4 (2); 0.3–8.2	2.1 (1); 0.05–11.1	2.3 (2); 0.3–8.0
Fatal, % (*n*)	0	0	0	0	0
Life-threatening, % (*n*)	2.1 (3)	1.7 (2)	2.4 (2)	0	2.3 (2)
ICH, % (*n*)	1.4 (2)	0	0	0	0
Serious bleeding events over 28 days^2^					
All events, % (*n*); 95% CI	9.2 (13); 5.0–15.2	5.9 (7); 2.4–11.7	7.1 (6); 2.6–14.7	4.2 (2); 0.1–14.3	6.8 (6); 2.5–14.3
Fatal, % (*n*)	0.7 (1)	1.7 (2)	1.2 (1)	2.1 (1)	2.3 (2)
Life-threatening, % (*n*)	2.1 (3)	3.4 (4)	4.7 (4)	0	3.4 (3)
ICH, % (*n*)	3.5 (5)	2.5 (3)	2.4 (2)	2.1 (1)	2.3 (2)
14-day mortality					
Mortality, % (*n*); 95% CI	14.1 (20); 8.8–20.9	10.1 (12); 5.3–17.0	9.4 (8); 4.2–17.7	8.3 (4); 2.3–20.0	10.2 (9); 4.8–18.5

^1^Patients lost to follow-up (no PF, MEN, or MD = 1; PF, MEN, or MD = 2) were excluded from this analysis; ^2^duration of follow-up for the open-label and compassionate-use studies was 28 days, and follow-up for the phase 2b open-label study was 14 days. DrotAA, drotrecogin alfa (activated); ICH, intracranial hemorrhage; MD, meningococcal disease; MEN, meningitis; PF, purpura fulminans.

**Table 7:** Characteristics of DrotAA-treated pediatric patients with PF, MEN, or MD and experienced a serious bleeding event

Characteristic	During infusion			After infusion			
		
Day of event^1^	1	1	7	8	10	UD	UD
Bleeding event							
ICH	No	No	No	Yes	No	Yes	Yes
Fatal	No	No	No	Yes	No	Yes	No
DrotAA related^2^	No	No	No	Yes	No	No	No
Disease category							
PF	Yes	Yes	Yes	Yes	Yes	No	Yes
MEN	No	Yes	No	No	No	Yes	No
MD	Yes	Yes	Yes	Yes	Yes	Yes	No
Baseline characteristics							
Protein C level (%)	36	-	16	41	-	34	-
Platelet count	26	23	33	30	68	71	14
APTT (s)	68	165	121	220	73	111	-
PT (s)	23	38	22	-	-	17	16
Organ failure	3	4	3	4	1	-	-

^1^Day 0 is defined as the calendar day on which DrotAA treatment began; ^2^This denotes whether or not the investigator considered the bleeding event to be related to treatment with DrotAA. APTT, activated partial thromboplastin time; BL, baseline; DrotAA, drotrecogin alfa (activated); ICH, intracranial hemorrhage; PT, prothrombin time; UD, unknown day after infusion. A dash indicates missing data.
